# *KCTD11* Tumor Suppressor Gene Expression Is Reduced in Prostate Adenocarcinoma

**DOI:** 10.1155/2014/380398

**Published:** 2014-06-19

**Authors:** Francesca Zazzeroni, Daniela Nicosia, Alessandra Tessitore, Rita Gallo, Daniela Verzella, Mariafausta Fischietti, Davide Vecchiotti, Luca Ventura, Daria Capece, Alberto Gulino, Edoardo Alesse

**Affiliations:** ^1^Department of Biotechnological and Applied Clinical Sciences, University of L'Aquila, Via Vetoio, Coppito 2, 67100 L'Aquila, Italy; ^2^Department of Pathology, San Salvatore Hospital, 67100 L'Aquila, Italy; ^3^Department of Molecular Medicine, “Sapienza” University of Rome, Via Regina Elena 291, 00161 Rome, Italy

## Abstract

Prostate cancer is the most common noncutaneous cancer among men in the United States. A genetic contribution to prostate cancer risk has been documented, but knowledge of the molecular mechanisms involved in prostate cancer initiation is still not well understood. Loss of heterozygosity (LOH) of chromosomal regions is crucial in tumor progression. In human prostate cancer, several chromosomal regions demonstrating a high frequency of LOH have been previously identified. *KCTD11 (REN)* is a tumor suppressor gene mapping on human chromosome 17p13.2, whose expression is frequently lost in human medulloblastoma and in several other cancer types. KCTD11 acts as a negative regulator of the Hedgehog (Hh) signaling. Here, we demonstrated that *KCTD11* LOH is a common genetic lesion in human prostate adenocarcinoma. Indeed, nuclear KCTD11 protein expression is strongly reduced in primary prostate cancer, and this event correlated with overexpression of proteins acting into the Hedgehog pathway. Low levels of *KCTD11* mRNA have been also observed in prostatic cancer cells, and ectopic overexpression of KCTD11 led to growth arrest. Our study demonstrates and supports that KCTD11, as well as negatively regulated downstream effectors belonging to Hh signaling, plays a role in prostate cancer pathogenesis. This could be suitable to characterize new diagnostic and therapeutic markers.

## 1. Introduction

Prostate cancer (CaP) is the most common noncutaneous cancer among men in the United States. The American Cancer Society estimated approximately 240.000 new diagnosed cases and 30.000 deaths due to this neoplasm in 2013 [1]. The introduction in clinical practice of PSA in the 1980s has influenced prostate cancer incidence, by permitting early diagnosis in some patients before symptoms develop or before abnormalities on physical examination are detectable [2]. The three most important risk factors are age, race, and family history. A genetic contribution to prostate cancer risk has been documented, but knowledge of the molecular mechanisms involved in prostate cancer initiation is still not well understood. In fact, carcinogenesis of prostate epithelial cells results from a complex series of initiation and progression events under environmental and genetic factors [3]. Sequence variants in several genes, such as SRD5A (steroid-5-alpha-reductase alpha polypeptide) [4], androgen receptor [5], estrogen receptor-*β* [6], E-cadherin [7], and toll-like receptors [8–10], have been associated with prostate cancer. Fusion of TMPRSS2 and ETS transcription factors genes occurs in approximately 50% of prostate cancer patients [11]. Moreover, BRCA1/2 [12], MMR genes [13], and HOXB13 [14] show potential clinical relevance in prostate cancer risk. Some studies showed LOH and chromosomal aberrations in prostate tumors [15–17]. Among these, Saric et al. demonstrated that the microsatellite D17S960 marker on the 17p13 chromosomal region is more markedly subjected to LOH in primary prostate carcinoma (43%) with respect to prostatic intraepithelial neoplasia (PIN) (18%) cases [15].

*KCTD11 (REN)* was originally identified by our group as a murine gene, playing a role in neural progenitor cell growth arrest and differentiation [18, 19]. Importantly,* KCTD11* has been further characterized as a tumor suppressor gene mapping on human chromosome 17p13.2, whose expression is frequently lost in human medulloblastoma and in several other cancer types due to both LOH and epigenetic events [20, 21]. In addition, it has been demonstrated that KCTD11 acts as a negative regulator of the Hedgehog (Hh) signaling in human medulloblastoma [20, 22].

Hedgehog signaling plays a key role in stem cell plasticity and in many developmental, physiological, and pathogenic processes [23]. Binding of the Hedgehog ligand to the Patched 1 (Patch1) receptor triggers a cascade of intracellular signaling activations that leads to the binding of downstream transcription factors (Gli1, Gli2, and Gli3) to their target sequences and then to the expression of target genes involved in the control of cell division or differentiation [24]. Aberrant Hh signaling activation has been implicated in prostate tumorigenesis in both human individuals and mouse models [25–32].

Here, we demonstrated that* KCTD11* LOH is a common genetic lesion also in human prostate adenocarcinoma. Indeed, nuclear KCTD11 protein expression is strongly decreased in primary prostate cancer, and this event is correlated to overexpression of proteins acting into the Sonic Hedgehog pathway. KCTD11 expression in prostatic cancer cells was also quite low, and ectopic overexpression of KCTD11 determined growth arrest through cyclin-dependent kinase inhibitors' upregulation and Hedgehog/Gli target genes' downregulation.

## 2. Materials and Methods

### 2.1. Prostate Adenocarcinoma Tissue Samples

Prostate Cancer-Normal Tissue Array (CA3) was purchased by SuperBioChips Tissue Array (Tema Ricerca Srl).

Eleven formalin-fixed, paraffin-embedded (FFPE) prostate adenocarcinoma tissue samples with Gleason between 5 and 10 and their matched normal counterparts were provided by the Pathology Unit of San Salvatore Hospital of L'Aquila. Tissue samples were microdissected in order to analyze just tumoral cells. Work was conducted in accordance with the Declaration of Helsinki.

### 2.2. DNA Extraction and LOH Analysis

Genomic DNA was extracted by using the Dinamite tissue kit (Labogen sas), according to the manufacturer's instructions. The microsatellite marker LOH analysis was performed by using the following primers: D17S960 FW 5′-TGATGCATATACATGCGTG-3′; D17S960REV 5′-TAGCGACTCTTCTGGCA-3′ (UniSTS:70862); D4S174 FW 5′-AAGAACCATGCGATACGACT-3′; D4S174 REV 5′-CATTCCTAGATGGGTAAAGC-3′ (UniSTS:3637). The reverse primers were ^32^P-labeled at 5′-end by T4 polynucleotide kinase. PCR was performed by using 100 ng of genomic DNA, 0.4 *μ*M each primer, 5% DMSO, and Taq DNA polymerase 2.5 U. PCR products were run onto a denaturing 5% polyacrylamide gel (7 M urea).

### 2.3. Cell Cultures

ALVA31 human prostate cancer cell line and Phoenix Ampho packaging cells were cultured in DMEM and supplemented with 10% FBS. Human prostate cancer cell lines PC3 and TSU were cultured in RPMI 1640 and supplemented with 10% FBS. LnCAP were cultured in RPMI 1640 medium containing 20% FBS, HEPES 1 mM, and glucose 4.5 g/L. All media were supplemented with glutamine 2 mM, streptomicyn 100 U/mL, and penicillin 100 U/mL.

### 2.4. Constructs, Transfections, and Transduction

The bicistronic retroviral construct MIGR1, expressing GFP, was obtained by MSCV vector [33]. MIGR-human KCTD11 was obtained by inserting a 1.5 Kb EcoRI fragment from pcRII-human KCTD11. Transfection of Phoenix Ampho packaging cells and infection of prostatic cancer cell lines were performed as previously described [33]. Infection efficiency was monitored by flow cytometry (FCM) and fluorescence microscopy. pCXN2-human KCTD11 [22] was transfected by using Fugene HD (Promega) according to the manufacturer's specifications. Transfection efficiency was monitored by flow cytometry (FCM) and fluorescence microscopy.

### 2.5. RNA Extraction and Q-RT-PCR

RNA was extracted by the use of Trizol reagent (Life Technologies) according to the manufacturer's specifications and reverse transcribed using the GeneAmp Gold RNA PCR Reagent Kit (Life Technologies). cDNA were amplified by using the following primers: KCTD11 FW 5′-GACACCTTCCGAAGCCAACC-3′, KCTD11 REV 5′-CCACTGCCACACCAAAT-3′; GLI1 FW 5′-GTGAGCCTGAATCTGTGTATGA-3′, GLI1 REV 5′-TGTGCTCGCTGTTGATGT-3′; PATCH1 FW 5′-CAGAATGGGTCCACGACAAA-3′, PATCH1 REV 5′-GTAGAAAGGGAACTGGGCATAC-3′; IGF-2 FW 5′-GTGCTGCATTGCTGCTTAC-3′, IGF-2 REV 5′-GGGCCTGCTGAAGTAGAAG-3′; Cyclin D2 FW 5′-CTGTGTGCCACCGACTTTA-3′, and Cyclin D2 REV 5′-GCGAGCTCACTTCCTCATC-3′. Q-RT-PCR was run on an Mx3000P (Statagene). Brilliant SYBR Green QPCR master mix 1x (Statagene) was used (GAPDH endogenous control). Quantitative analysis was performed by Mx3000P software.

### 2.6. Proliferation Assay

MIGR-hKCTD11-infected prostate cancer cell lines were cultured onto a 12 mm diameter glass. After 24 hrs, BrdU was added (BrdU labeling and detection kit, Boehringer Mannheim) according to the manufacturer's instructions. Cells were incubated for 8 and for 24 hours and then fixed in 4% paraformaldehyde for 10 min, permeabilized with 0.25% Triton X in PBS, washed, and incubated with primary antibody (anti-GFP, Santa Cruz Biotechnology). After 1 hr, cells were washed and incubated with goat anti-rabbit Alexa Flour 488 (Santa Cruz Biotech) secondary antibody for 45 min. Cells were treated with 4% paraformaldehyde and then 2 N HCl. After three washes, cells were incubated with 1 : 10 primary anti-BrdU (BrdU labeling and detection kit, Boehringer Mannheim) and, afterward, with goat anti-mouse IgG TRITC (Sigma-Aldrich) secondary antibody. Hoechst was used for nuclear staining. Cells were analyzed by using a Zeiss Axioplan 2 (Carl Zeiss) fluorescence microscope.

### 2.7. Immunohistochemistry

Immunohistochemistry analysis was performed on tissue arrays from prostate cancers and normal tissues (SuperBioChips Tissue Array) and on FFPE prostate adenocarcinoma samples from the Unit of Pathology of L'Aquila San Salvatore hospital. Analysis was performed as previously described [21]. Primary antibodies were as follows: anti-*α*-REN antibody [18, 21], *α*-Gli1 (sc-6153, Santa Cruz Biotechnology), and *α*-Patch1 (sc-6149, Santa Cruz Biotechnology). All antibodies were diluted in ultrAb diluent (Lab Vision) and incubated overnight at 4°C. Tissues were analyzed by using a Nikon Eclipse E200 microscope. Negative controls were performed by omitting primary antibodies.

Immunostaining for KCTD11 was semiquantitatively scored as “**−**” (no or less than 5% positive cells), “+” (5–25% positive cells), “++” (26–50% positive cells), “+++” (51–75% positive cells), and “++++”(75–100% positive cells). Quantitative analysis was performed by counting positive cells in three different fields (magnification ×40).

### 2.8. Western Blot

Cell lysates were obtained as previously described [18]. Primary antibodies used were anti-p21 (mouse monoclonal, Calbiochem), anti-p27 (mouse monoclonal, Transduction Laboratories), anti-COOH1 [18, 21], and anti-actin (Santa Cruz Biotechnology). Secondary antibodies: anti-mouse-HRP, anti-rabbit-HRP, and anti-goat-HRP (Santa Cruz Biotechnology).

### 2.9. Statistical Analysis

Statistical analysis was performed using the unpaired 2-tailed Student's *t*-test. *P* values less than 0.05 were considered significant.

## 3. Results

### 3.1. *KCTD11* LOH in Prostate Adenocarcinoma

A previous work showed that LOH of microsatellite D17S960 marker occurs in 18% of prostatic intraepithelial neoplasia (PIN), 43% of primary, and 57% of metastatic prostate cancer (CaP) lesions [15], thus identifying the loss of tumor suppressor genes on chromosomal arm 17p as an early event in CaP evolution. The authors suggested that p53 and hypermethylated in carcinoma 1 (HIC1) genes as tumor suppressors were potentially deleted because they were located close to the D17S960 chromosomal locus.

**Figure 1:**
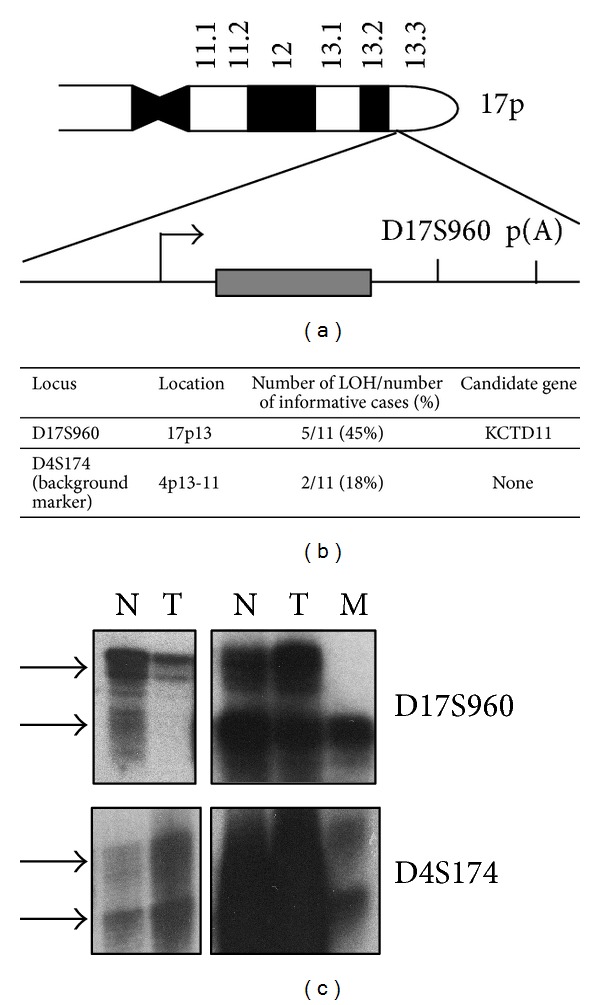
*KCTD11* loss of heterozygosity (LOH) analysis in prostate cancer samples matched with paired normal tissues. (a) Schematic representation of human* KCTD11* locus. D17S960 microsatellite marker position is shown. (b)* KCTD11* LOH frequency in prostate adenocarcinoma samples (D17S960, specific microsatellite marker, D4S174, background marker). (c) Representative images of* KCTD11* LOH in prostate adenocarcinoma (N normal tissues, T tumoral tissue, and M metastasis).

As shown in Figure 1(a) and as previously described [20], microsatellite D17S960 marker is set at the 3′-UTR of* KCTD11* single-exon tumor suppressor gene. To determine the role of* KCTD11* gene in CaP, we first decided to seek for LOH of* KCTD11* in 11 human primary CaP tissues. Tumoral and normal samples were obtained from the same FFPE tissue block by microdissection. LOH analysis revealed that 45% of our cases showed allelic deletion (Figures 1(b) and 1(c)). LOH of microsatellite D4S174 was used as a background marker. This result is in accordance with the data of Saric et al. [15], demonstrating that* KCTD11* deletion represents a genetic alteration of CaP.

### 3.2. KCTD11 Expression Is Downregulated in Prostate Adenocarcinoma

**Figure 2:**
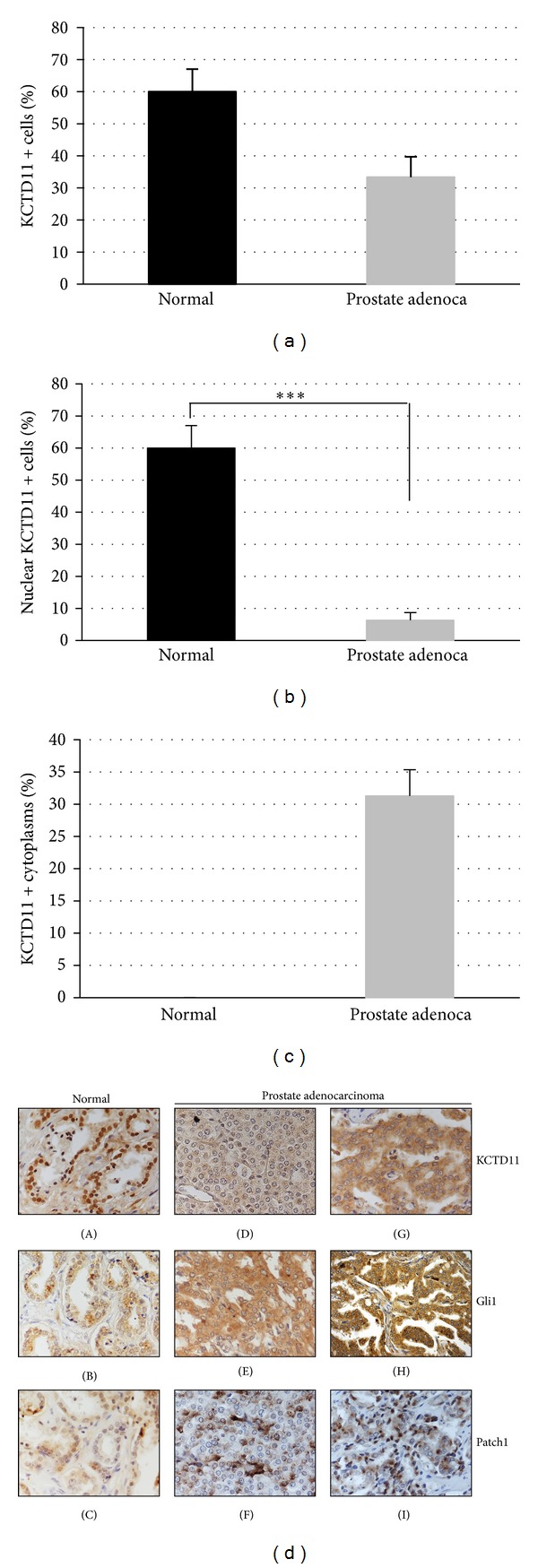
KCTD11 expression in prostate cancer cells compared to normal cells. (a) Total KCTD11-positive cells in normal and prostate adenocarcinoma tissues. (b) Nuclear KCTD11-positive cells in normal and prostate adenocarcinoma tissues. (***P < 0.005) (c) Cytoplasmic KCTD11-positive cells in normal and prostate adenocarcinoma tissues. ((a)–(c)) Data are mean ± S.D. (d) KCTD11, Gli1, and Patch1 expressions in normal ((A), (B), and (C)) and prostate adenocarcinoma ((D)–(I)). Magnification 40x.

To extend our analysis at protein level, a commercially available Prostate Cancer-Normal Tissue Array containing 40 samples of prostate adenocarcinoma and 7 samples of normal prostatic tissue was analyzed for KCTD11 expression. Diagnosis, Gleason, and stage of each tissue were provided by the manufacturer (http://www.tissue-array.com/) and reported in Table 1. Normal prostate epithelial cells showed a high nuclear expression of KCTD11 (Table 1) with a positive cells mean corresponding to 60% (Figures 2(a) and 2(b) and Figure 2(d) panel (A)), suggesting a role for this gene in prostate physiology. On the contrary, in CaP tissues KCTD11 protein expression was found to be reduced (Table 1 and Figure 2(a)), with only 32% of prostatic cells expressing KCTD11. Notably, in most CaP tissues KCTD11 expression was completely lost (Table 1 and Figure 2(d) panel (D)) and in those tumoral tissues showing positivity KCTD11 expression was observed mainly in the cytoplasm (Figures 2(c) and 2(d) panel (G)). Indeed, considering just nuclear KCTD11-positive cells, CaP tissues showed a strong and significant reduction of expression of this tumor suppressor gene (Figure 2(b)).

### 3.3. Reduced Expression of KCTD11 Correlates with Increased Expression of Sonic Hedgehog Signaling Proteins

KCTD11 was previously identified as a suppressor of Hedgehog signaling [20, 22], and deregulation of this pathway has been extensively implicated in prostate tumorigenesis [25–31]. Therefore, contextually to KCTD11, we analyzed the expression level of Patch1 and Gli1 in the same CaP tissues setting. As shown in Figure 2(d), normal prostate epithelial cells showed low expression levels of both Patch1 and Gli1 (Figure 2(d) panels (B) and (C)), whereas in prostate cancers, in which KCTD11 was either lost or expressed in the cytoplasm, both Patch1 and Gli1 resulted to be overexpressed (Figure 2(d) panels (E)-(F), (H)-(I)).

### 3.4. KCTD11 Inhibits Prostate Cell Proliferation* In Vitro*

**Figure 3:**
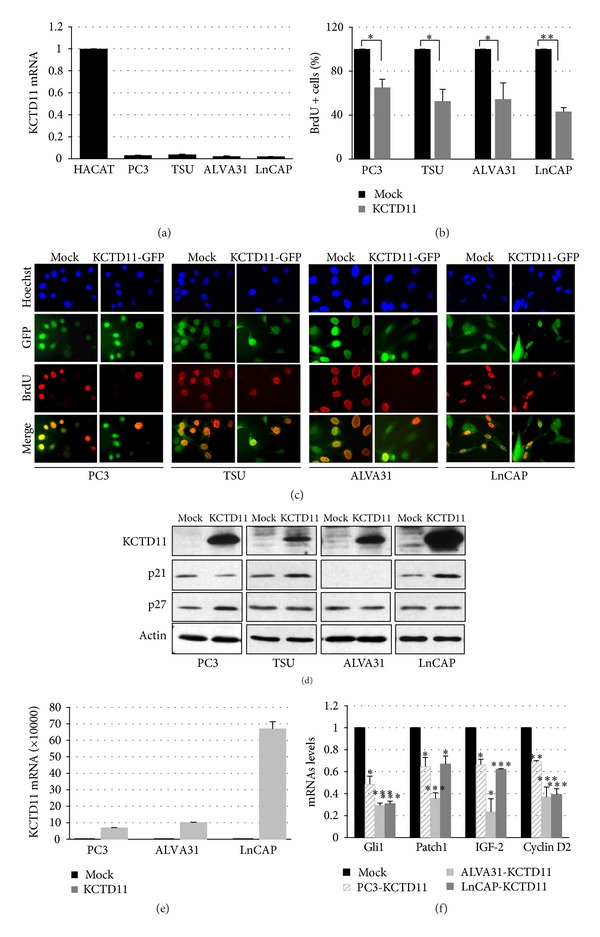
KCTD11 inhibits cellular proliferation. (a) Q-RT-PCR analysis of* KCTD11* mRNA expression in prostate cancer cell lines. (GAPDH was used as endogenous control.) Data are mean ± S.D. of triplicate wells. (b) BrdU-positive cells in KCTD11 overexpressing cell lines. Data are mean ± S.D. from three replicates (*P < 0.05; **P < 0.01). (c) Representative images of BrdU immunofluorescence assay in prostatic cells stably transfected with KCTD11-GFP. (d) Western blot showing p21 and p27 expression levels in prostate cell lines overexpressing KCTD11. (e) Q-RT-PCR analysis of* KCTD11* mRNA expression in pCXN2-human KCTD11 transfected prostate cancer cell lines. (GAPDH was used as endogenous control.) Data are mean ± S.D. of triplicate wells. (f) Q-RT-PCR analysis of* Gli1, Patch1, IGF-2, and Cyclin D2* mRNA expressions in KCTD11 overexpressing cells. Data are mean ± S.D. of triplicate wells. (*P < 0.05; **P < 0.01, and ***P < 0.005.)

It has been previously shown that KCTD11 inhibits cell proliferation [18–20]. To clarify the role of KCTD11 gene in prostatic cell growth, we analyzed PC3, TSU, ALVA31, and LnCAP prostate cell lines. Firstly,* KCTD11* basal expression was assessed in these cell lines, showing significantly low levels of this transcript (Figure 3(a)). This data demonstrated that KCTD11 downregulation occurs also in prostatic cell lines. As reference, HACAT cell line showing high KCTD11 expression levels was used. Next, we generated KCTD11-GFP-overexpressing prostate cell lines by retroviral infection. High expression levels of KCTD11-GFP were confirmed both by flow cytometry analysis [data not shown] and western blot (Figure 3(d), upper panel). The effect of KCTD11 overexpression on cell proliferation was measured by BrdU incorporation (Figures 3(b) and 3(c)). All tested cell lines showed a decrease in cell growth ranging from 40 to 60%. Growth inhibition correlated with upregulation of cyclin-dependent kinase inhibitors p21^WAF1^ and p27^KIP1^ (Figure 3(d)). In addition, KCTD11 overexpressing prostate cancer cells (Figure 3(e)) displayed a reduction of cell proliferation-related genes, which are known direct targets of Hedgehog/Gli1, such as Cyclin D2 and IGF-2 (Figure 3(f)). Moreover, a decrease of both Patch1 and Gli1 was also observed (Figure 3(f)). Together, these data indicated that KCTD11 upregulation is necessary for inhibiting cell proliferation in prostate cells, through its ability to upregulate cyclin-dependent kinase inhibitors and downregulate Hedgehog/Gli target genes.

## 4. Discussion

Although prostate cancer is associated with a longer natural history than most other tumor types and most of the men diagnosed with prostate cancer every year do not die from the disease, it remains the second leading cause of cancer death in men [1]. Despite an improved understanding of prostate tumor biology, the reason why certain prostate tumors behave more aggressively than others is still not clear. Furthermore, all patients with prostate cancer are treated similarly once metastases have developed, and patients vary widely in their response to therapies. Understanding the molecular alterations driving prostate cancer can aid in the development of new biomarkers as well as therapeutic targets.

Substantial progress has been made in the last decade to understand the genetic landscape of prostatic cancer. For example, one of the most common genetic alterations discovered is the fusion of TMPRSS2 with ETS gene family of transcription factors [11]. These gene fusions occur at the early stage of the disease pathogenesis, being present in high-grade prostate intraepithelial cancer [34, 35]. In addition, they are prostate-specific and therefore not present in benign prostatic hyperplasia or any other tumor type. This discovery has major implications in prostate cancer diagnosis. In fact, the TMPRSS2-ERG fusion is now being prospectively evaluated as a diagnostic urinary test to complement prostate-specific antigen (PSA) screening [36]. Several other genetic lesions have been identified in prostate cancer, such as SRD5A (steroid-5-alpha-reductase alpha polypeptide) [4], androgen receptor [5], estrogen receptor-*β* [6], E-cadherin [7], and toll-like receptors [8–10].

Loss of heterozygosity (LOH) of chromosomal regions is crucial in tumor progression. In human prostate cancer, several chromosomal regions demonstrating a high frequency of LOH have been previously identified. LOH has been reported on chromosomes 1p, 3p, 5q, 7q, 6q, 8p, 8q, 10q, 11p-q, 13q, 16q, 17p, 17q, 18q, and 19p [15–17].

Human* KCTD11 (REN)* has been described as a novel tumor suppressor gene located in the short arm of chromosome 17, at the 17p13.2 locus.* KCTD11 (REN)* was isolated by our group as a murine immediate-early gene induced by neurogenic stimuli (NGF, EGF, and retinoic acid) in pluripotent embryonal stem cells and neural progenitor cell lines [18].* KCTD11* upregulation has been associated with neurotypic differentiation, growth arrest, and p27^Kip1^ induction [17, 18]. In addition, its expression is tightly regulated during development. In fact,* KCTD11* is strongly expressed in E7.5-E8.5 mouse embryo with levels decreasing thereafter and it localizes preferentially to neuroectodermal cells [18].

Importantly, the human orthologue of murine* KCTD11* has been shown to be frequently lost in human medulloblastoma (MB). In fact,* KCTD11 *LOH occurs in 39% of human sporadic MB. Furthermore, all diploid and hemizygous MBs showed a strong reduction of* KCTD11* expression, suggesting that silencing of this gene is a pivotal event in MB tumorigenesis [20]. Notably,* KCTD11* expression is frequently downregulated in several human cancers, including larynx, esophagus, stomach, colon-rectum, urinary bladder, lung, breast, gallbladder, and endometrium and its promoter was found to be methylated in cancer [21]. Regarding its molecular activity,* KCTD11* has been shown to encode for a novel adaptor of Cullin3 ubiquitin E3 ligase complex targeting histone deacetylase 1 and able to inhibit the Hedgehog signaling pathway [20, 22]. Gli1 and Gli2 are transcription factors activated by Hedgehog signaling. They are acetylated proteins and their HDAC-mediated deacetylation promotes transcriptional activation and sustains a positive autoregulatory loop through Hedgehog-induced upregulation of HDAC1. This mechanism is turned off by HDAC1 degradation through an E3 ubiquitin ligase complex formed by Cullin3 and KCTD11 [22]. In this work, we identified* KCTD11* as a gene frequently lost in prostate cancer, showing both LOH and decreased protein expression. Our data are in accordance with KCTD11 LOH data from the Cancer Genome Atlas (TCGA) resource database (http://www.cbioportal.org/), in which deletion of KCTD11 gene is reported to occur in prostate adenocarcinoma. In most CaP tissues, KCTD11 nuclear expression was completely lost, whereas LOH was observed only in 45% of analyzed tissues. Previous work from our group demonstrated that epigenetic events, such as methylation, downregulate KCTD11 in cancer [21]. Moreover, in around 30% of prostate tumoral tissues, KCTD11 expression was observed mainly in the cytoplasm, suggesting that in these cases the protein could be mutated or sequestered by a deregulated protein interactor.

Notably, KCTD11 downregulation is associated with Gli1 and Patch1 overexpression, demonstrating that* KCTD11* tumor suppressor gene acts as an inhibitor of Hedgehog signaling not only in medulloblastoma [20] but also in prostate cancer. Moreover, aberrant Hh signaling activation has been implicated in prostate tumorigenesis in both human subjects and mouse models [25–32], and preclinical data have shown that inhibition of Hh signaling has the potential to reduce prostate cancer invasiveness and metastasis spreading [33]. However, acquired drug resistance has already been described in other cancers upon long-term treatment with Hh inhibitors. Therefore, the identification of factors involved in Hh signaling regulation, which are subjected to alterations and/or genetic lesions, might open a new scenario for designing new target therapies in prostate cancer.

## 5. Conclusions

Prostate cancer is one of the most common malignant diseases among men in developed countries, which has become a major public health challenge. Traditionally considered as a disease of elderly men, an increasing proportion of prostate cancer cases now occur in men of preretirement ages. New markers for identifying high-risk populations as well as novel strategies for early detection and preventive care are urgently needed.

The mechanism of prostate tumorigenesis is still not fully understood. Here, we showed both LOH and decreased protein expression of the tumor suppressor gene* KCTD11* in prostate cancer, which is associated with increased expression of Hh proteins. KCTD11 expression in prostate cancer cells was also quite low, and ectopic overexpression of KCTD11 determined growth arrest through cyclin-dependent kinase inhibitors upregulation and Hedgehog/Gli target genes' downregulation. Taken together, these data suggest that KCTD11 can be considered a potential candidate to be used for diagnostic and therapeutic application in prostate cancer.

## Figures and Tables

**Table 1:** KCTD11 expression in prostate adenocarcinoma tissues.

No.	Diagnosis	Gleason	Stage	KCTD11 expression	KCTD11 nuclear expression
1	Adenocarcinoma	9	III	++	−
2	Adenocarcinoma	7	II	−	−
3	Adenocarcinoma	9	III	−	−
4	Adenocarcinoma	10	III	+	−
5	Adenocarcinoma	9	III	−	−
6	Adenocarcinoma	8	IV	−	−
7	Adenocarcinoma	7	II	−	−
8	Adenocarcinoma	7	II	+	−
9	Adenocarcinoma	7	II	+++	+
10	Adenocarcinoma	9	III	+++	−
11	Adenocarcinoma	9	III	−	−
12	Adenocarcinoma	7	III	−	−
13	Adenocarcinoma	7	IV	−	−
14	Adenocarcinoma	9	III	−	−
15	Adenocarcinoma	9	IV	−	−
16	Adenocarcinoma	7	III	++++	++
17	Adenocarcinoma	9	IV	−	−
18	Adenocarcinoma	7	III	+++	+
19	Adenocarcinoma	7	III	−	−
20	Adenocarcinoma	9	III	++	−
21	Adenocarcinoma	7	III	++	+
22	Adenocarcinoma	7	II	++	−
23	Adenocarcinoma	7	III	−	−
24	Adenocarcinoma	6	II	−	−
25	Adenocarcinoma	9	III	++	−
26	Adenocarcinoma	9	III	+	−
27	Adenocarcinoma	8	III	−	−
28	Adenocarcinoma	6	III	++++	−
29	Adenocarcinoma	7	II	++++	+
30	Adenocarcinoma	8	III	++++	+
31	Adenocarcinoma	10	IV	−	−
32	Adenocarcinoma	7	IV	−	−
33	Adenocarcinoma	8	IV	−	−
34	Adenocarcinoma	8	III	++++	−
35	Adenocarcinoma	9	III	+	−
36	Adenocarcinoma	9	IV	+	−
37	Adenocarcinoma	9	IV	−	−
38	Adenocarcinoma	9	IV	++++	−
39	Adenocarcinoma	8	III	++++	+
40	Adenocarcinoma	7	III	++++	+
42	Normal (match of #9)	—	—	+++	+++
43	Normal (match of #12)	—	—	+	+
44	Normal (match of #14)	—	—	+++	+++
45	Normal (match of #18)	—	—	++	++
47	Normal (match of #29)	—	—	+++	+++
48	Normal (match of #31)	—	—	+++	+++
49	Normal (match of #40)	—	—	++++	++++

List of prostate adenocarcinoma and normal tissues analyzed (SuperBioChips Tissue Array). Staging and grading of each sample were obtained from manufacturer (http://www.tissue-array.com/). Evaluation of total KCTD11 expression (5th column) or nuclear KCTD11 expression (6th column) in prostate adenocarcinoma tissues was shown. Scores were as follows: “−” <5%; “+” 1–25%; “++” 25–50%; “+++” 50–75%; “++++” 75–100%.
